# DNA methylation may mediate psychotropic drug-induced metabolic side effects: results from a 1-month observational study

**DOI:** 10.1192/j.eurpsy.2022.285

**Published:** 2022-09-01

**Authors:** C. Dubath, E. Porcu, A. Delacrétaz, C. Grosu, N. Laaboub, M. Piras, A. Von Gunten, P. Conus, K. Von Plessen, Z. Kutalik, C. Eap

**Affiliations:** 1 Lausanne University Hospital, Psychiatry, Prilly, Switzerland; 2 Swiss institute of bioinformatics, Bioinformatics, Lausanne, Switzerland

**Keywords:** psychotropic drugs, Metabolic side effects, Precision Medicine, epigenetics

## Abstract

**Introduction:**

Metabolic side effects of psychotropic medications are a major drawback to patients’ effective treatment. Among the mechanisms underlying their development, DNA methylation may be involved.

**Objectives:**

The aim of this study was to estimate DNA methylation changes occurring secondary to psychotropic treatment and evaluate associations between 1-month metabolic changes and baseline DNA methylation or 1-month DNA methylation changes, using an epigenome-wide approach.

**Methods:**

Seventy-nine psychiatric patients recruited as part of PsyMetab study, who started a treatment with either an antipsychotic, a mood stabilizer or mirtazapine were selected. Epigenome-wide DNA methylation was measured using the Illumina Methylation EPIC BeadChip at baseline and after one month of treatment.

**Results:**

A global methylation increase was observed after 1 month of treatment, which was more pronounced in patients whose weight remained stable (i.e., <2.5% weight increase). Epigenome-wide significant methylation changes were observed at 52 loci in the whole cohort and at one site, namely cg12209987, located in an intergenic region within an enhancer, specifically in patients who underwent important early weight gain (i.e., ≥5% weight increase) during the same period of treatment (p<5*10^-8^). Multivariable analysis confirmed an association between an increase in methylation at this locus and weight gain in the whole cohort (p=0.004). Epigenome-wide association analyses failed to identify any significant link between other metabolic changes (e.g. glucose or lipid levels) and methylation data.

**Conclusions:**

These findings give new insight into the mechanisms of psychotropic drug-induced weight gain. With improved understanding of the metabolic side effects, the use of precision medicine with epigenetics may become possible

**Disclosure:**

No significant relationships.

